# Supplementary material for: Chemopreventive Activity of Ellagitannins from Acer pseudosieboldianum (Pax) Komarov Leaves on Prostate Cancer Cells
Source: Plants (Basel). 2023 Feb 24;12(5):1047. doi: 10.3390/plants12051047 (PMC10005130; doi:10.3390/plants12051047)
Supplement: Supplementary file 1 [file plants-12-01047-s001.zip › plants-2217421-supplementary.pdf]

Supplementary Materials

Chemopreventive Activity of Ellagitannins from *Acer pseudosieboldianum* (Pax) Komarov Leaves on Prostate Cancer Cells

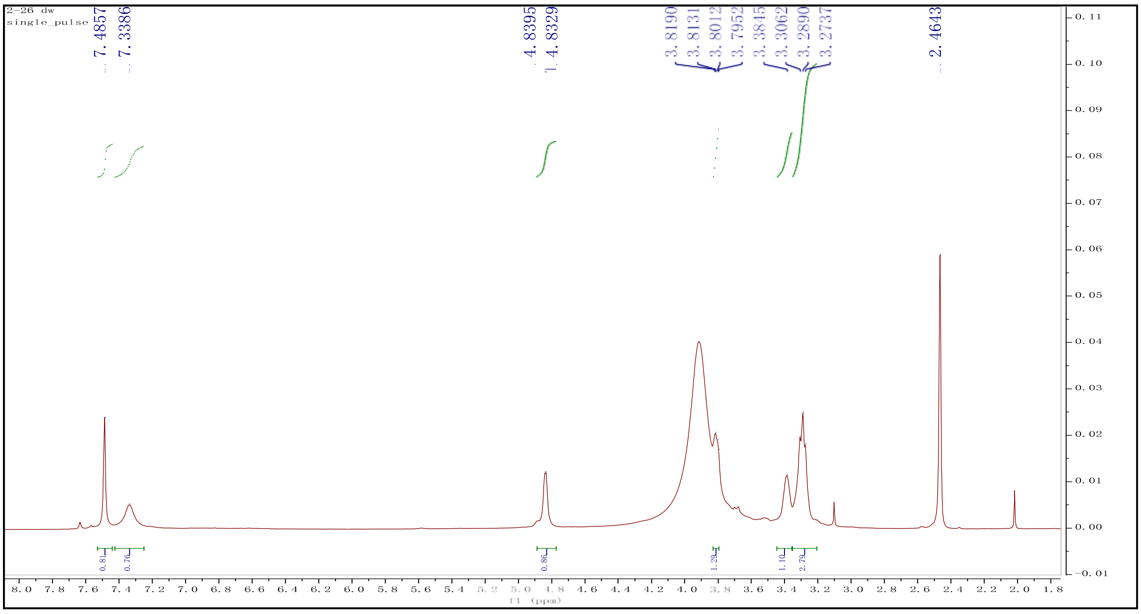

Figure S1. ^{1}H-NMR spectrum (in 600 MHz) of compound **1** in DMSO-d_6+ D_2O

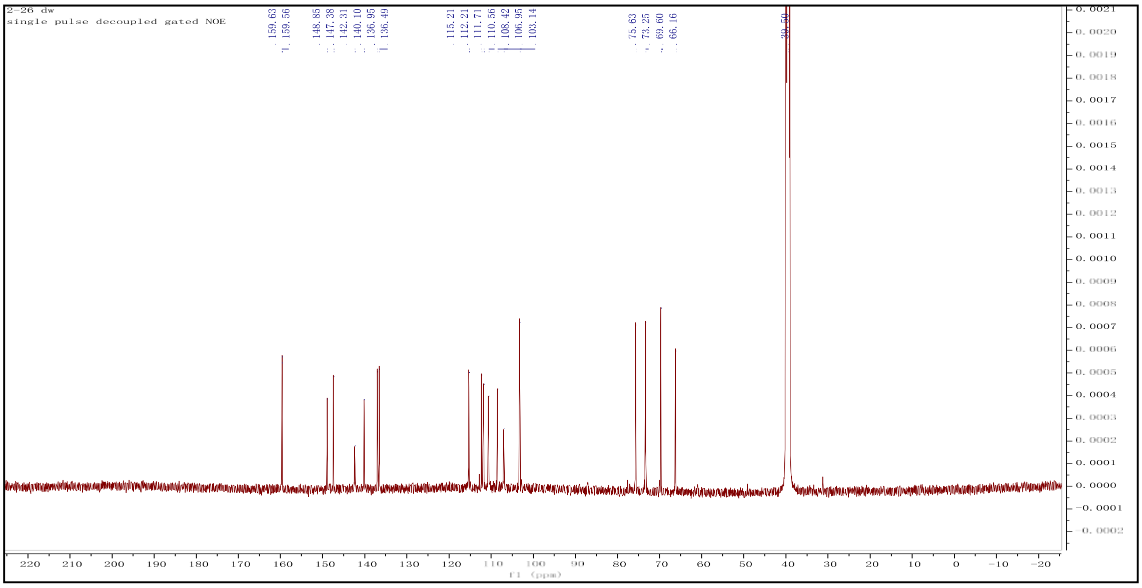

Figure S2. ^{13}C-NMR spectrum (in 150 MHz) of compound **1** in DMSO-d_6+ D_2O

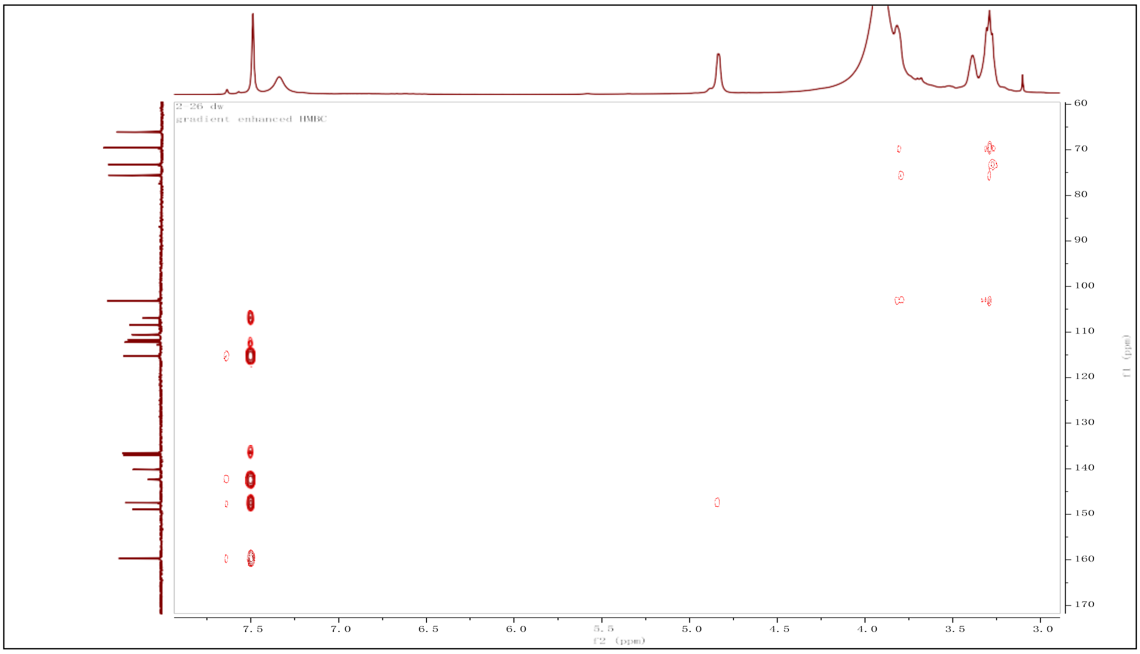

Figure S3. HMBC spectrum of compound **1** in DMSO-d_6+ D_2O

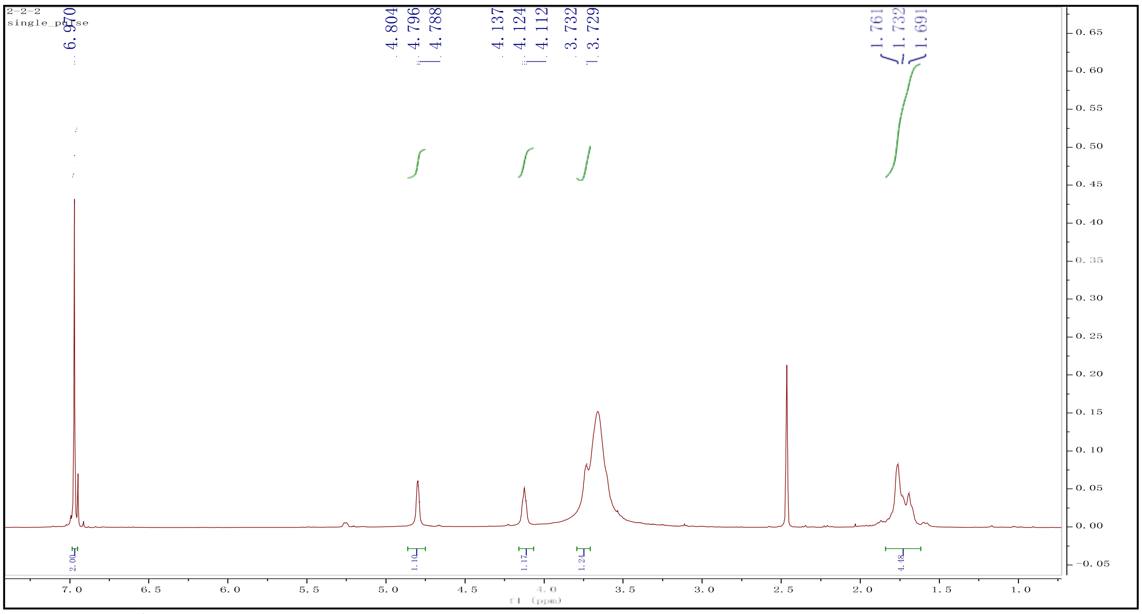

Figure S4. ^{1}H-NMR spectrum (in 600 MHz) of compound **2** in DMSO-d_6+ D_2O

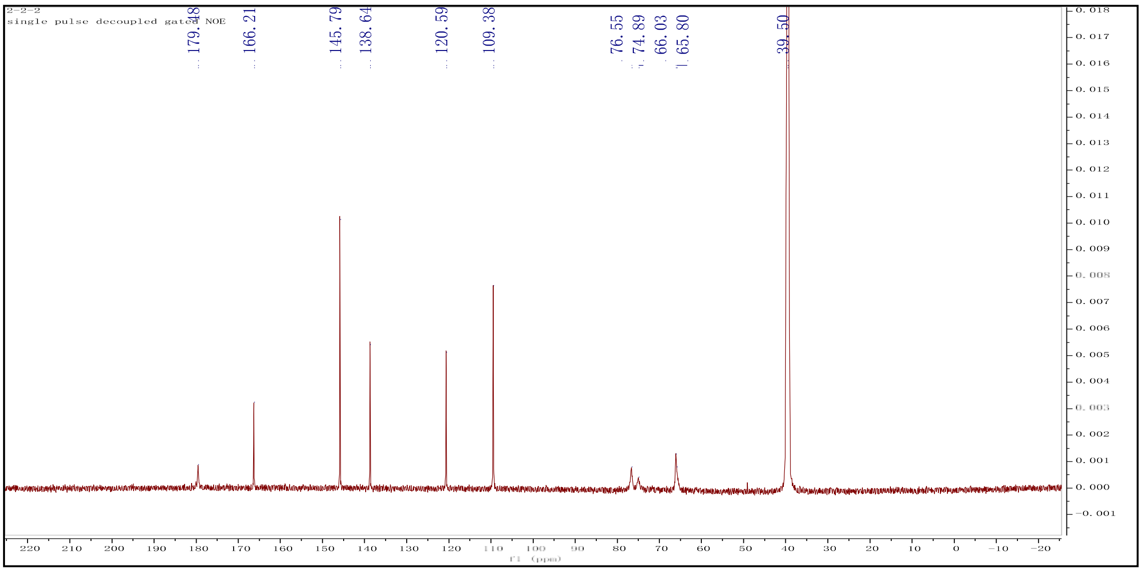

Figure S5. ^{13}C-NMR spectrum (in 150 MHz) of compound **2** in DMSO-d_6+ D_2O

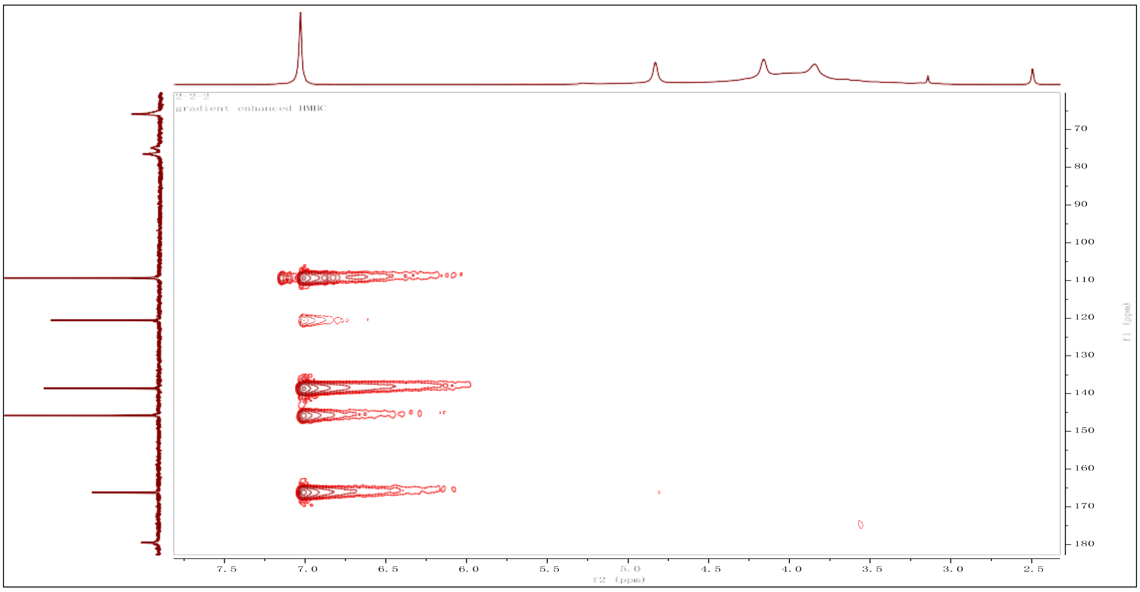

Figure S6. HMBC spectrum of compound **2** in DMSO-d_6+ D_2O

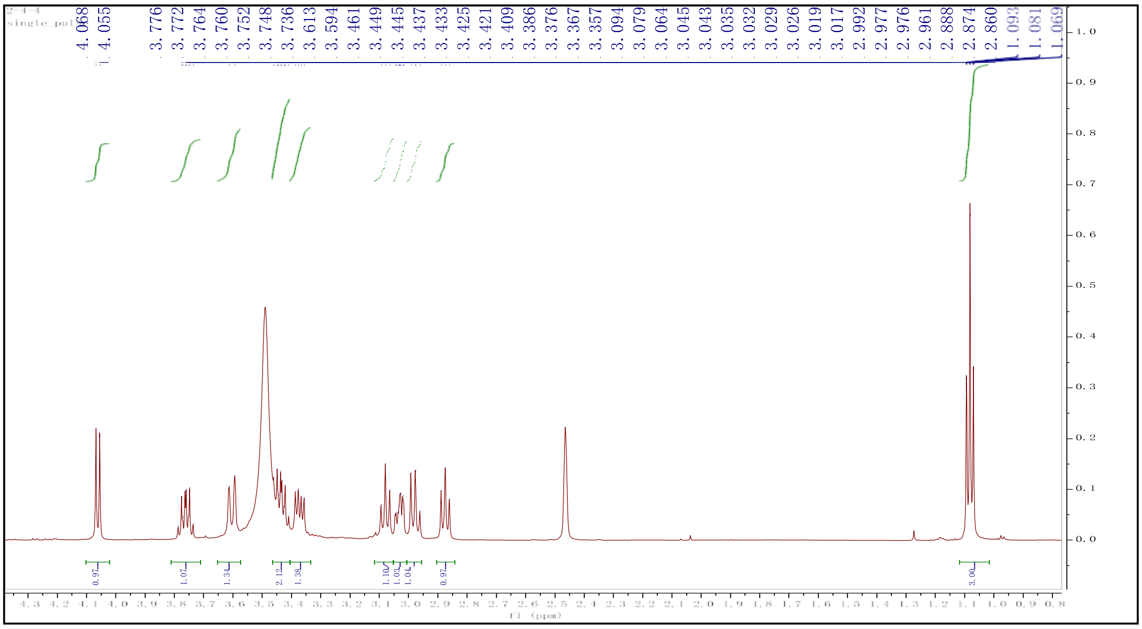

Figure S7. ^{1}H-NMR spectrum (in 600 MHz) of compound **3** in D_2O

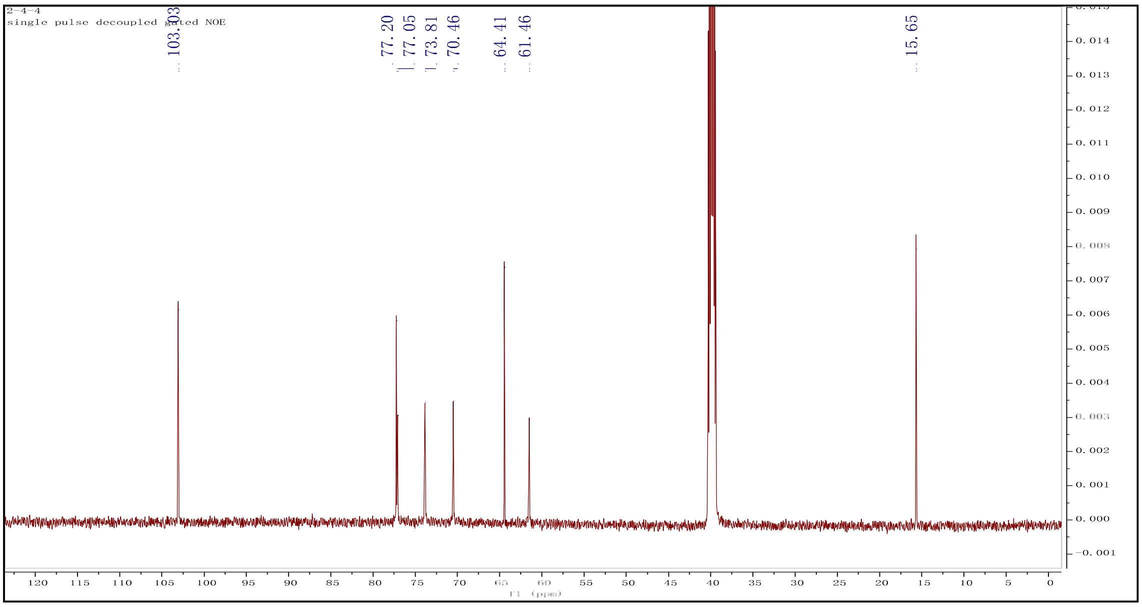

Figure S8. ^{13}C-NMR spectrum (in 150 MHz) of compound **3** in D_2O

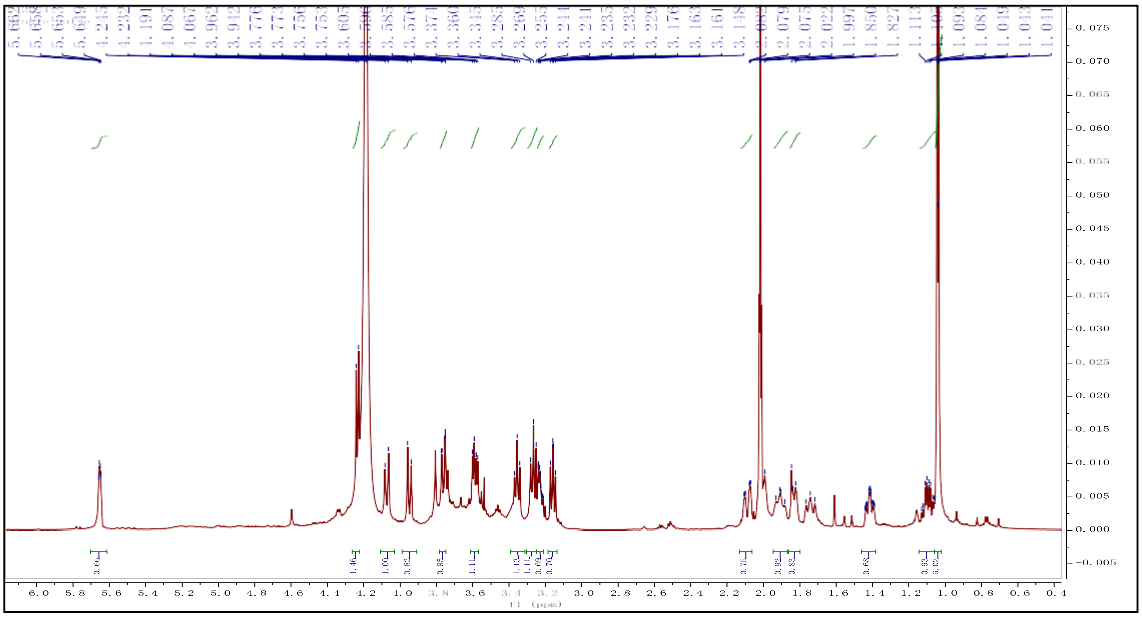

Figure S9. ^{1}H-NMR spectrum (in 600 MHz) of compound **4** in $(CD_3OD)_2CO$-d_6 + D_2O

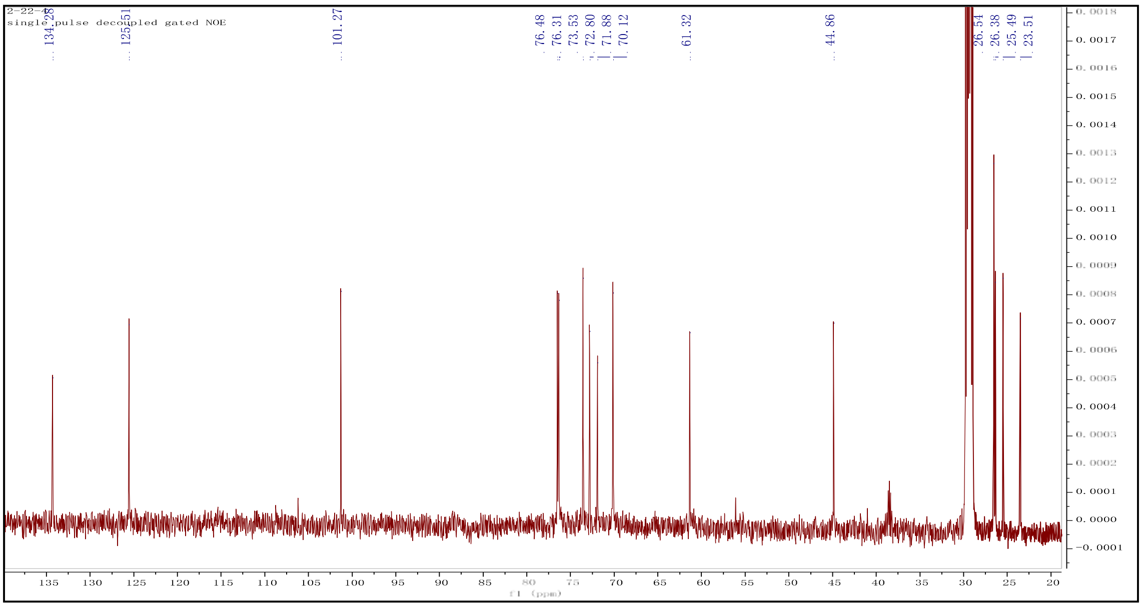

Figure S10. ^{13}C-NMR spectrum (in 150 MHz) of compound **4** in $(CD_3OD)_2CO$-d_6 + D_2O

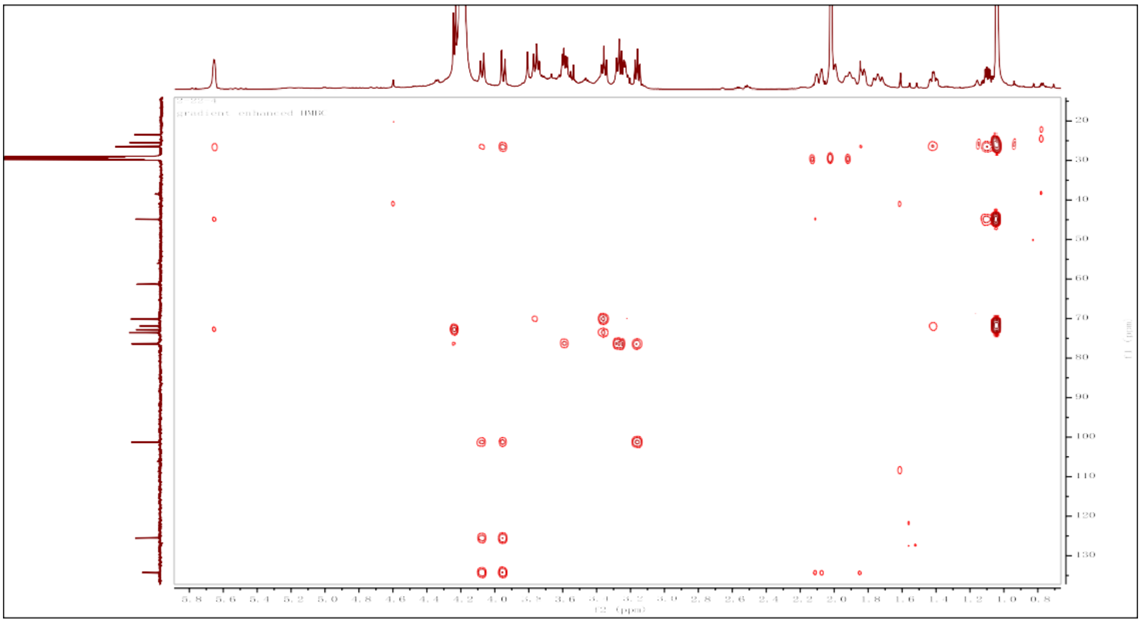

Figure S11. HMBC spectrum of compound **4** in $(CD_3OD)_2CO$- d_6 + D_2O

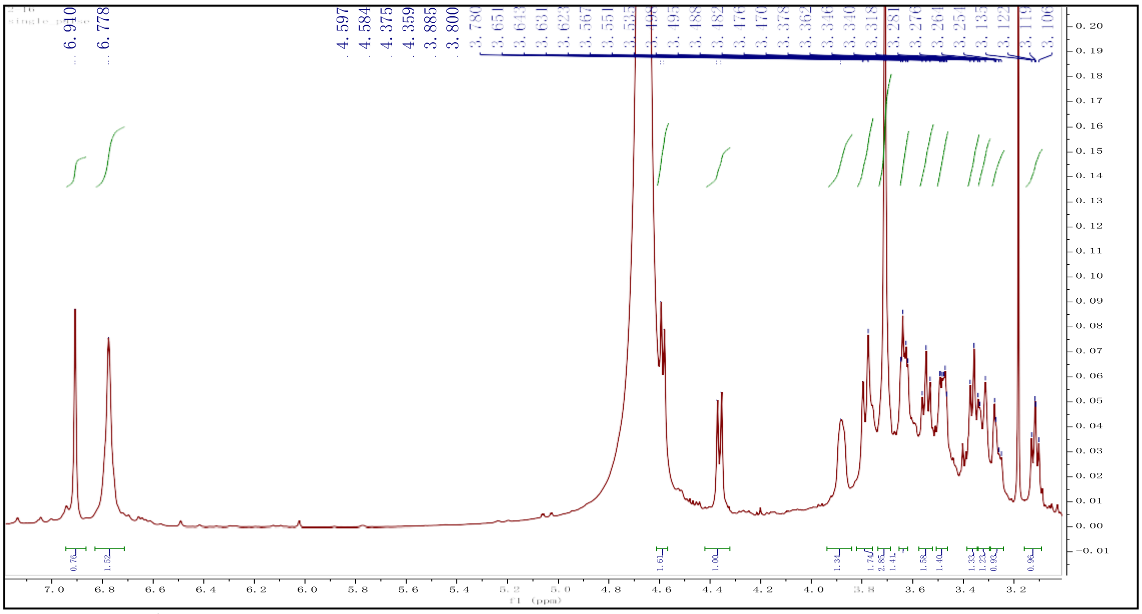

Figure S12. ^{1}H-NMR spectrum (in 600 MHz) of compound **5** in DMSO-d_6 + D_2O

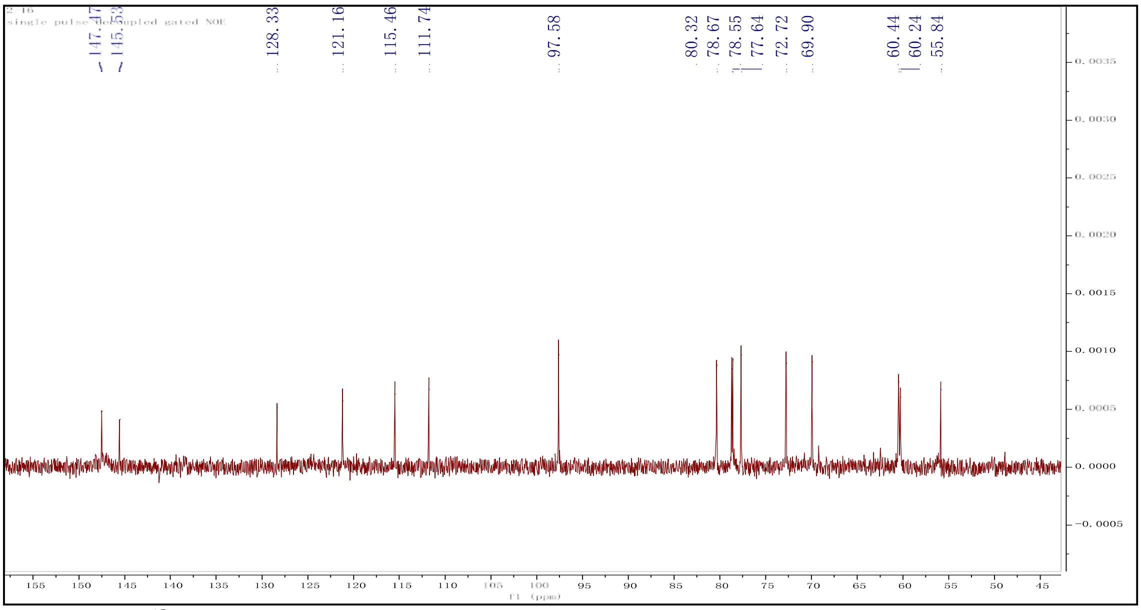

Figure S13. ^{13}C-NMR spectrum (in 150 MHz) of compound **5** in DMSO-d_6 + D_2O

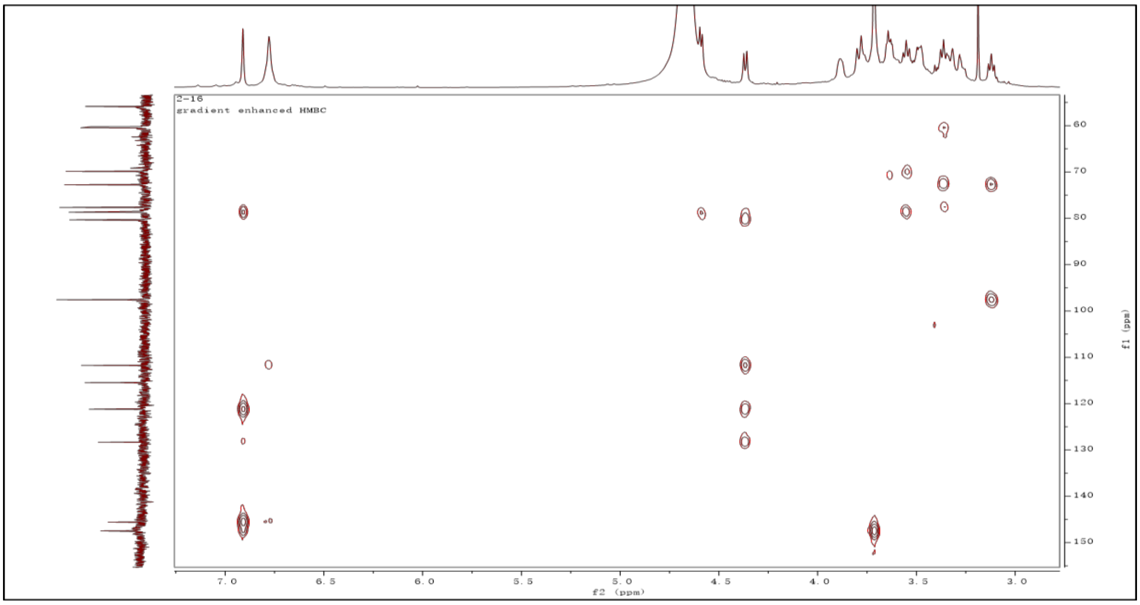

Figure S14. HMBC spectrum of compound **5** in $(CD_3OD)_2CO$- d_6 + D_2O

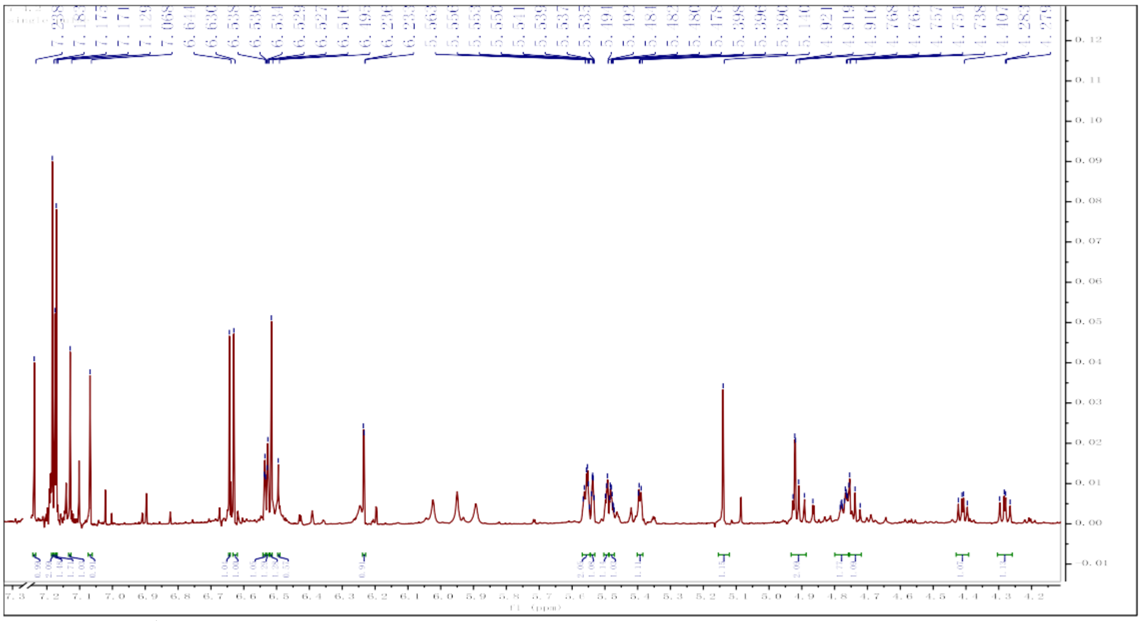

Figure S15. ^{1}H-NMR spectrum (in 600 MHz) of compound **6** in $(CD_3OD)_2CO$- d_6 + D_2O

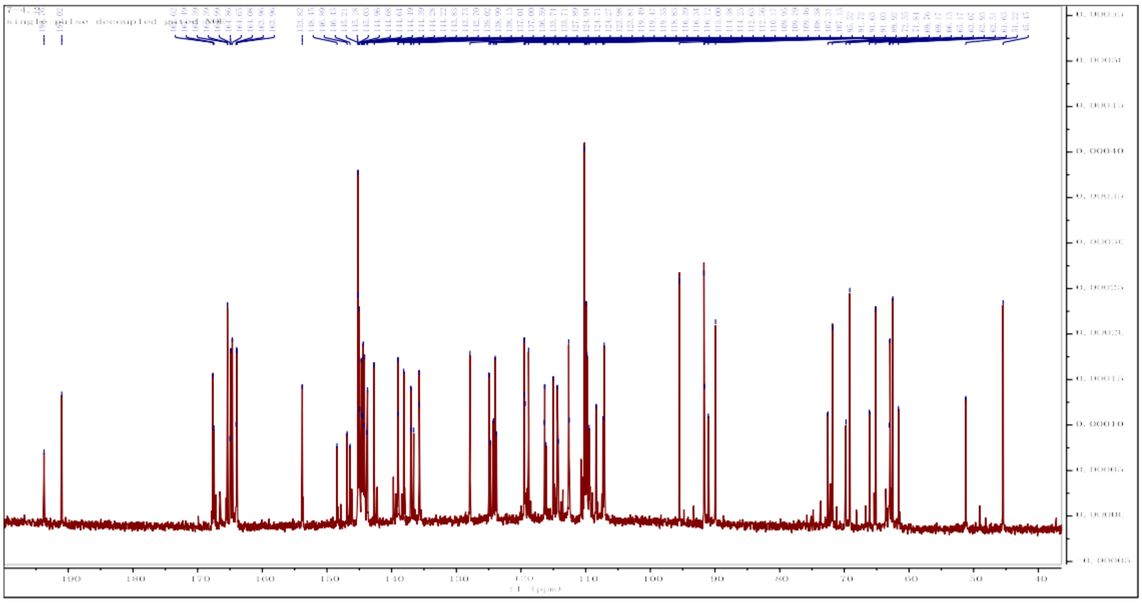

Figure S16. ^{13}C-NMR spectrum (in 150 MHz) of compound **6** in $(CD_3OD)_2CO$- d_6 + D_2O

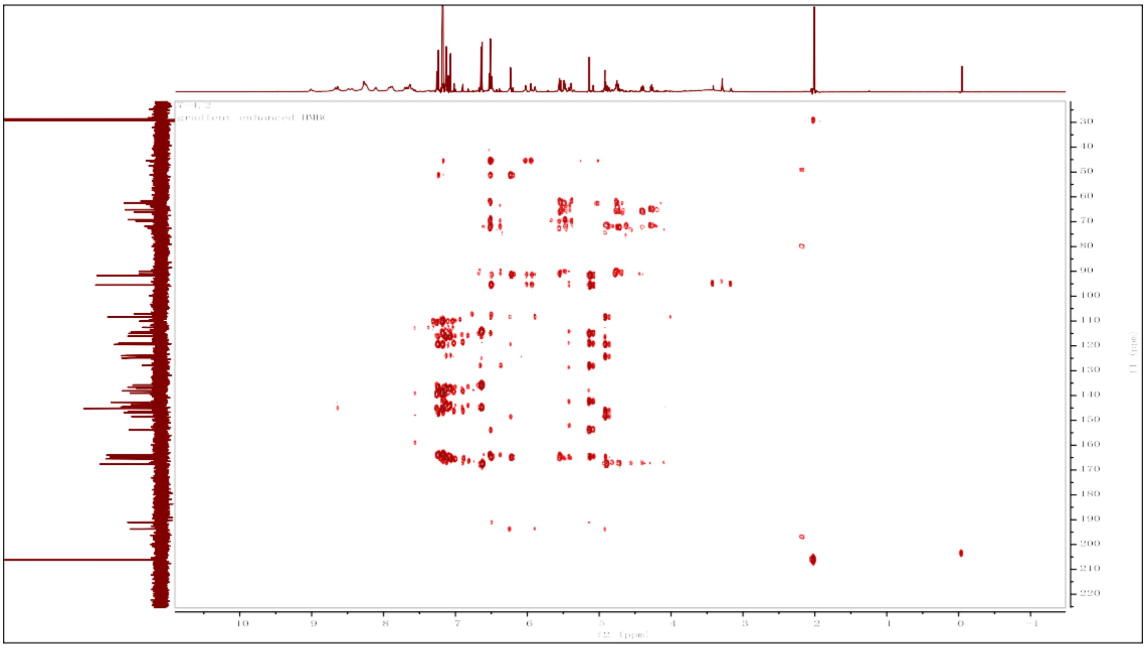

Figure S17. HMBC spectrum of compound **6** in $(CD_3OD)_2CO$- d_6 + D_2O

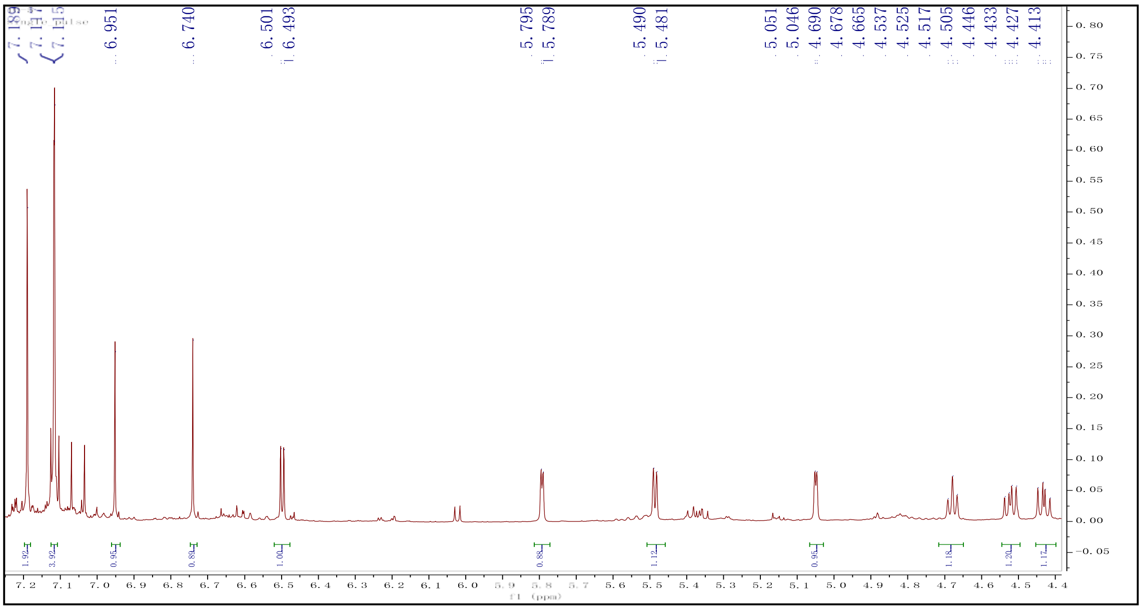

Figure S18. ^{1}H-NMR spectrum (in 600 MHz) of compound **7** in $(CD_3OD)_2CO$- d_6 + D_2O

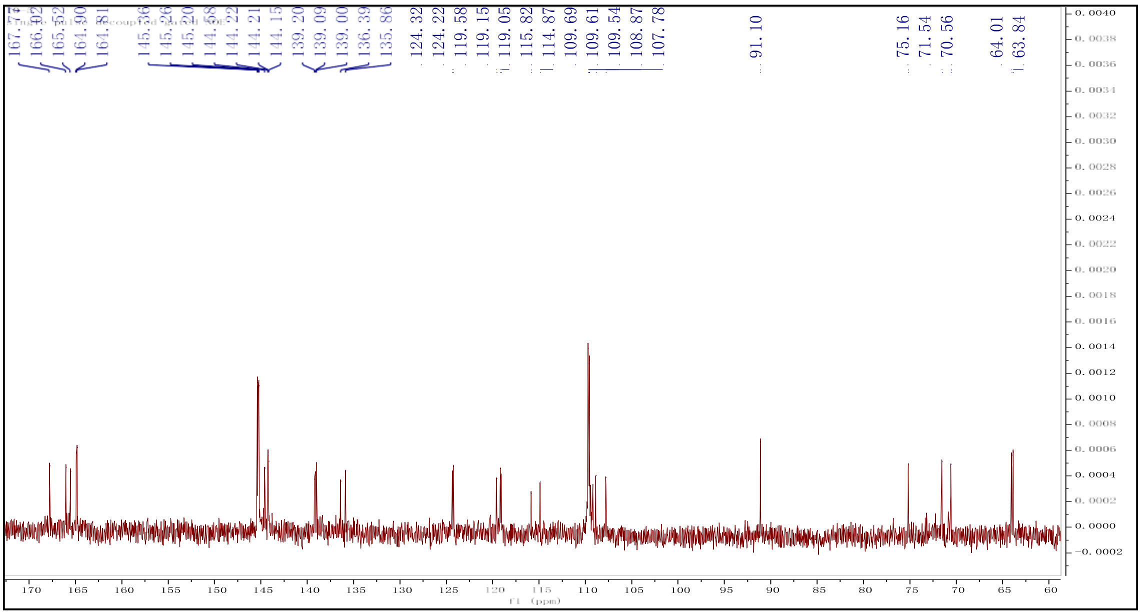

Figure S19. ^{13}C-NMR spectrum (in 150 MHz) of compound **7** in $(CD_3OD)_2CO$- d_6 + D_2O

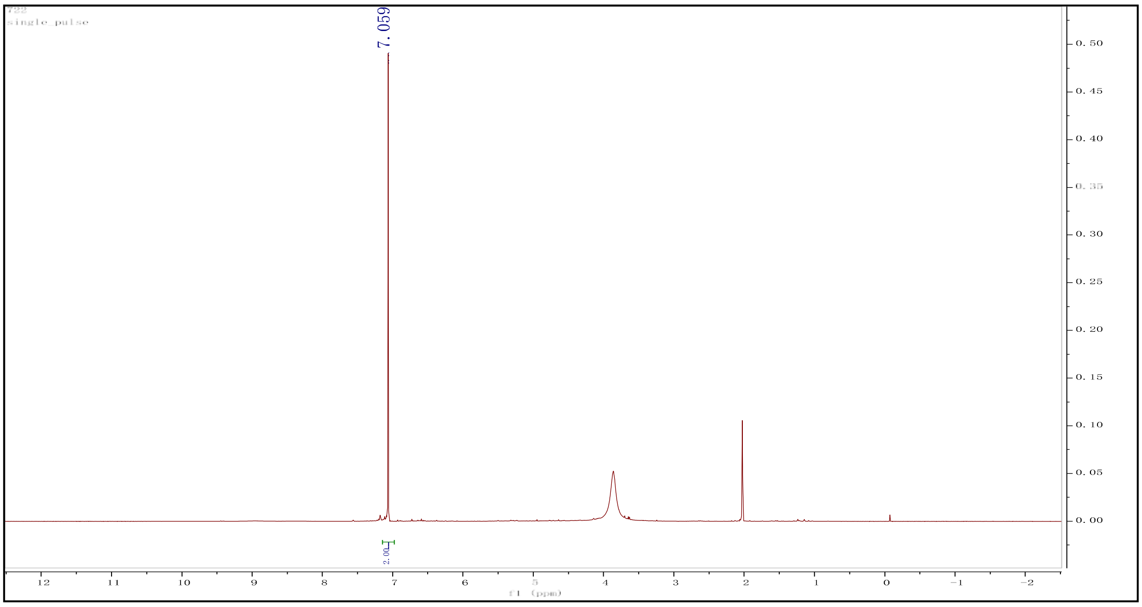

Figure S20. ^{1}H-NMR spectrum (in 600 MHz) of compound **8** in $(CD_3OD)_2CO$- d_6 + D_2O

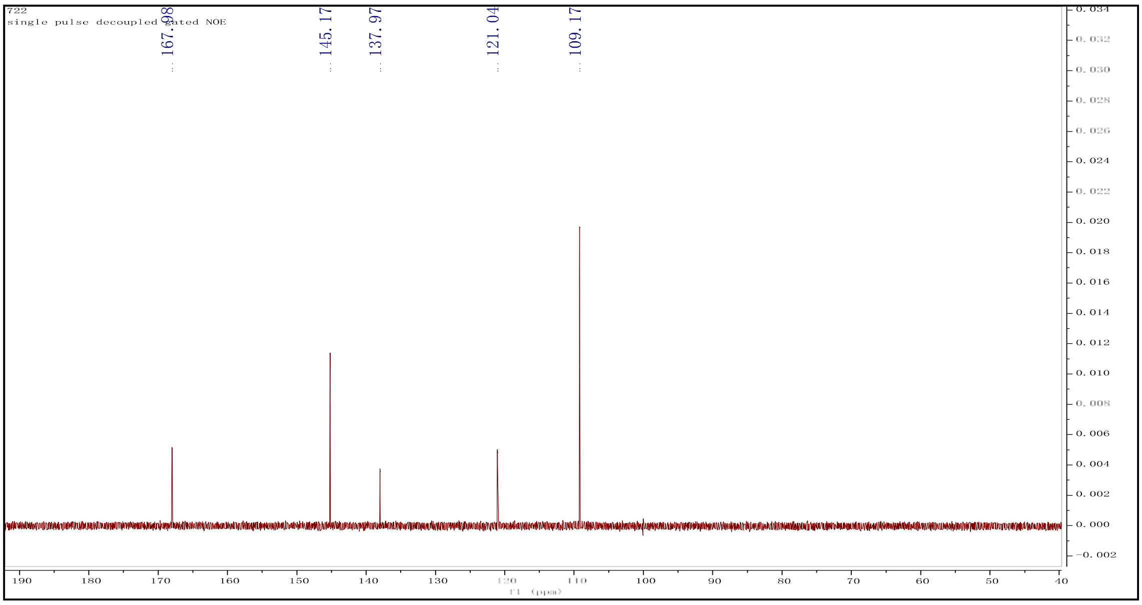

Figure S21. ^{13}C-NMR spectrum (in 150 MHz) of compound **8** in $(CD_3OD)_2CO$- d_6+ D_2O

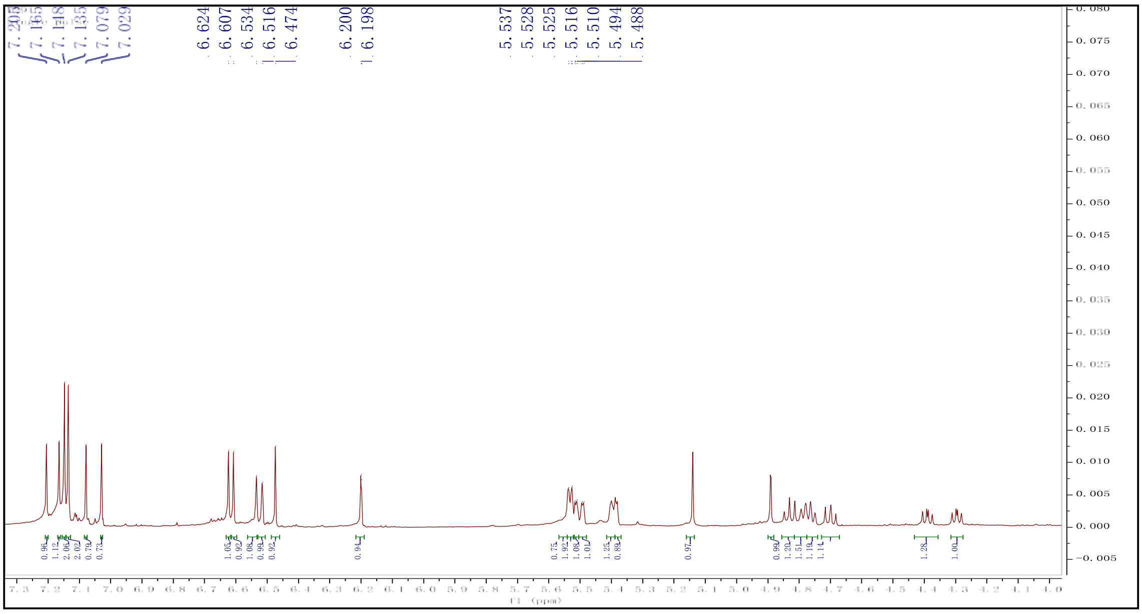

Figure S22. ^{1}H-NMR spectrum (in 600 MHz) of compound **9** in $(CD_3OD)_2CO$- d_6 + D_2O

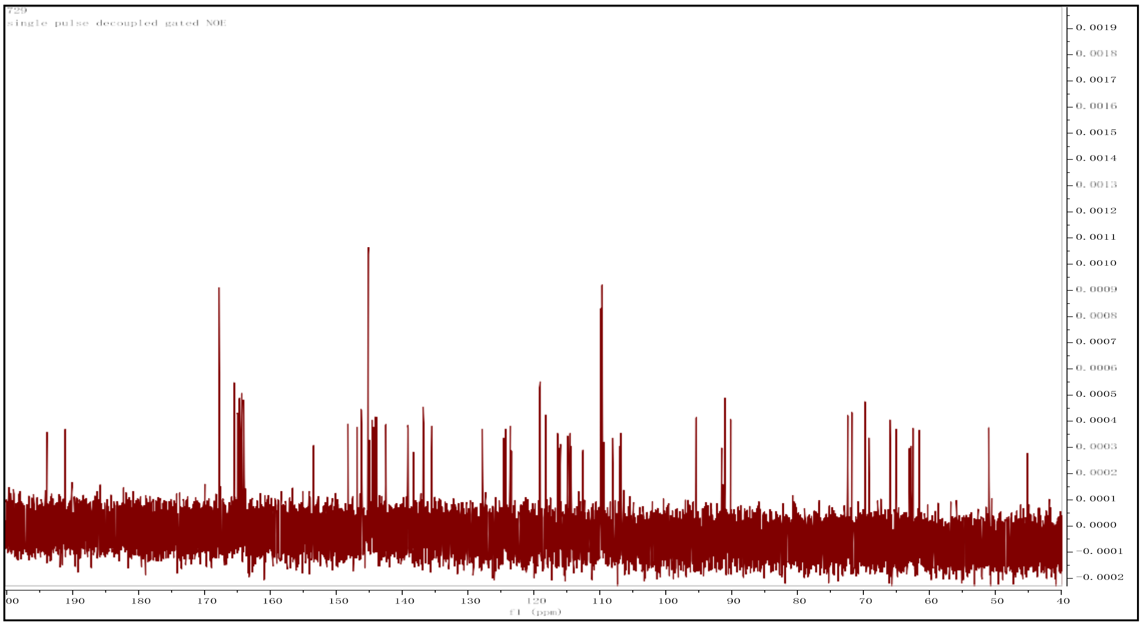

Figure S23. ^{13}C-NMR spectrum (in 150 MHz) of compound **9** in $(CD_3OD)_2CO$- d_6 + D_2O

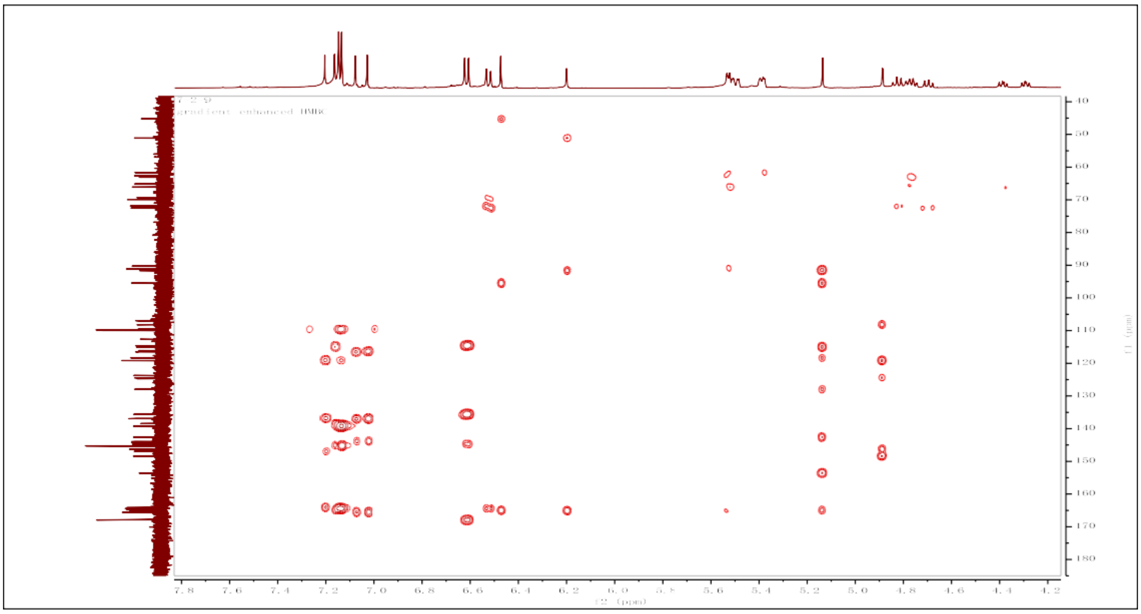

Figure S24. HMBC spectrum of compound **9** in $(CD_3OD)_2CO$- d_6+ D_2O

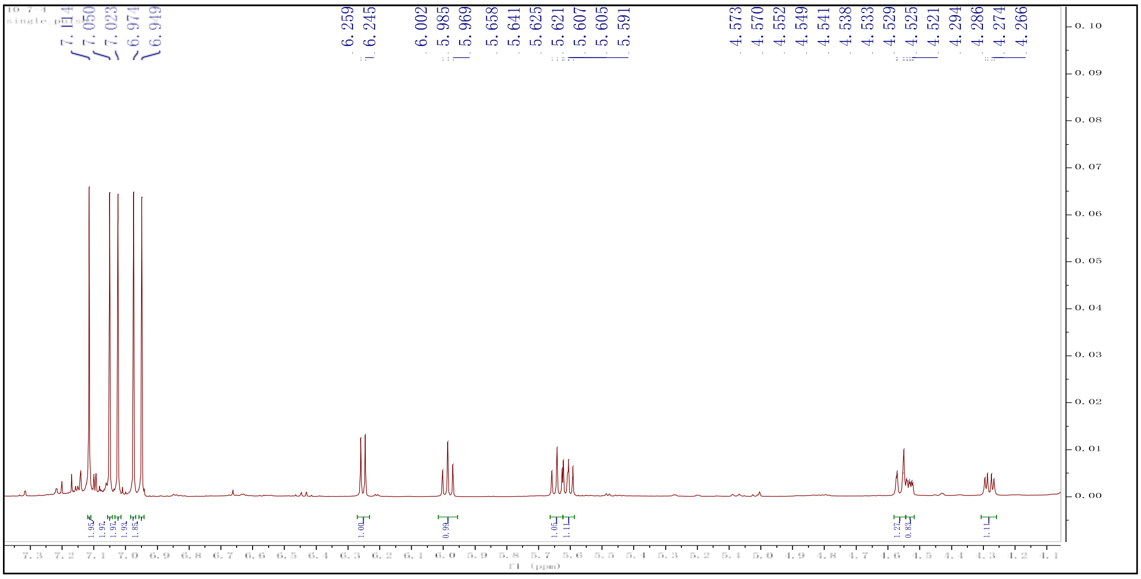

Figure S25. ^{1}H-NMR spectrum (in 600 MHz) of compound **10** in $(CD_3OD)_2CO$- d_6+ D_2O

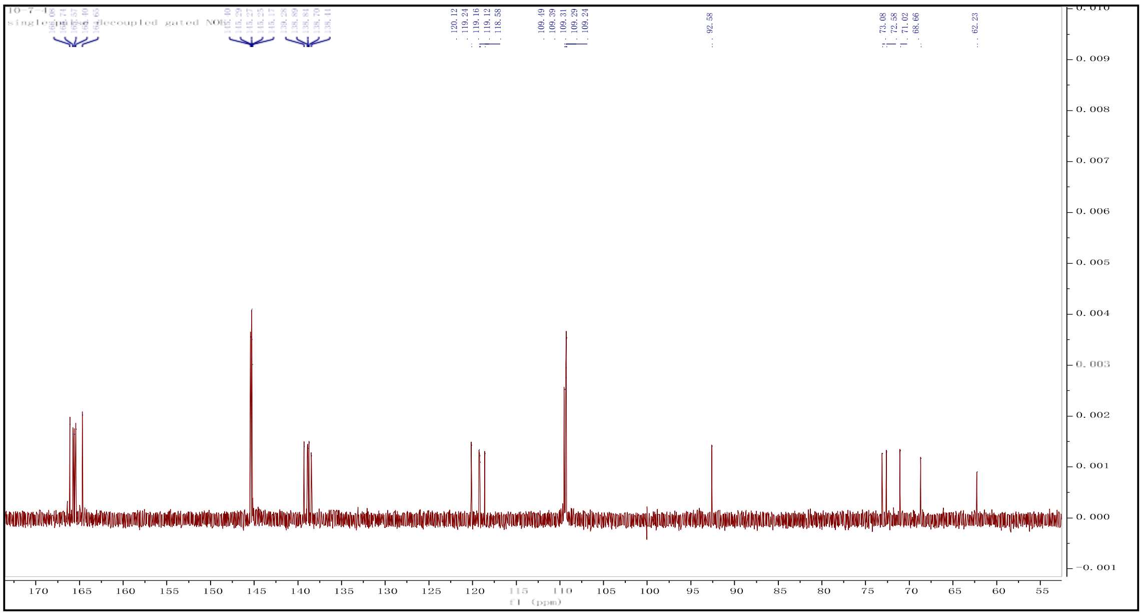

Figure S26. ^{13}C-NMR spectrum (in 150 MHz) of compound **10** in $(CD_3OD)_2CO$- d_6 + D_2O

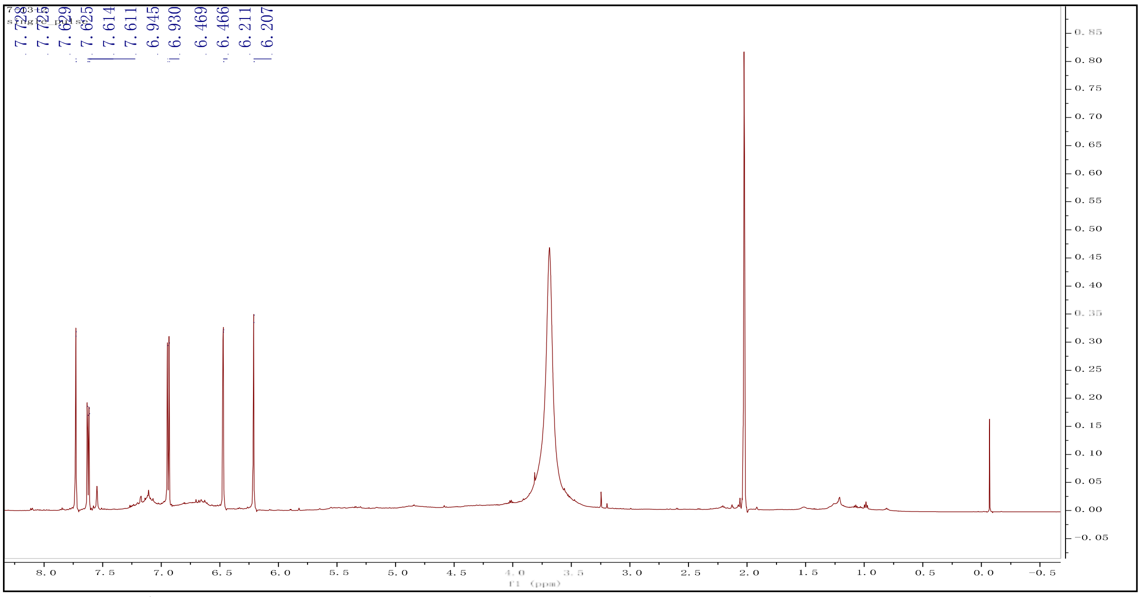

Figure S27.^{1}H-NMR spectrum (in 600 MHz) of compound **11** in $(CD_3OD)_2CO$- d_6 + D_2O

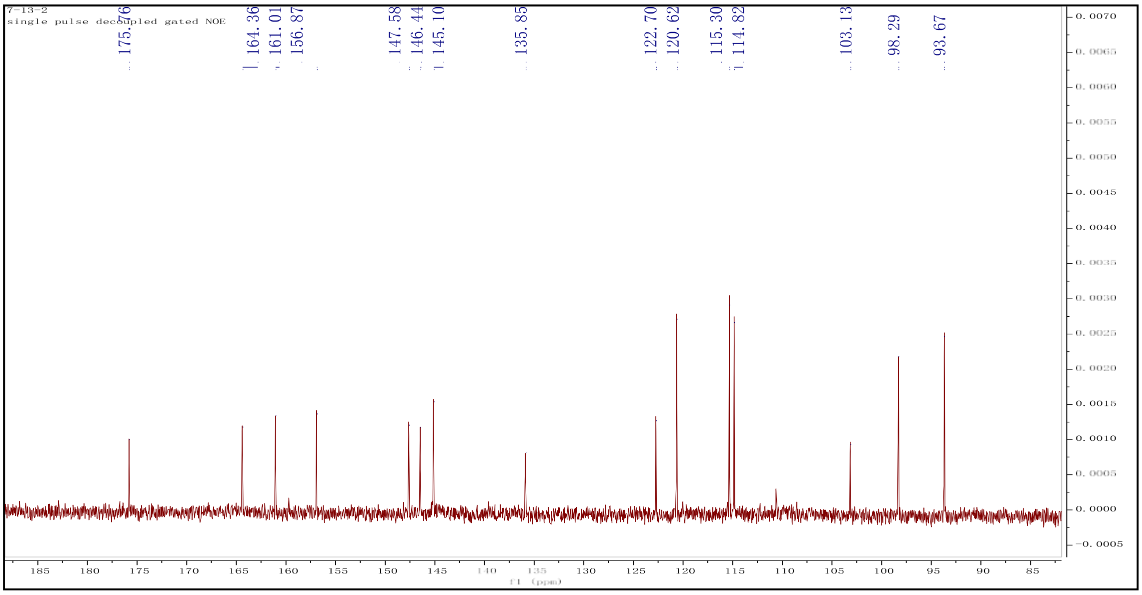

Figure S28.^{13}C-NMR spectrum (in 150 MHz) of compound **11** in $(CD_3OD)_2CO$- d_6 + D_2O

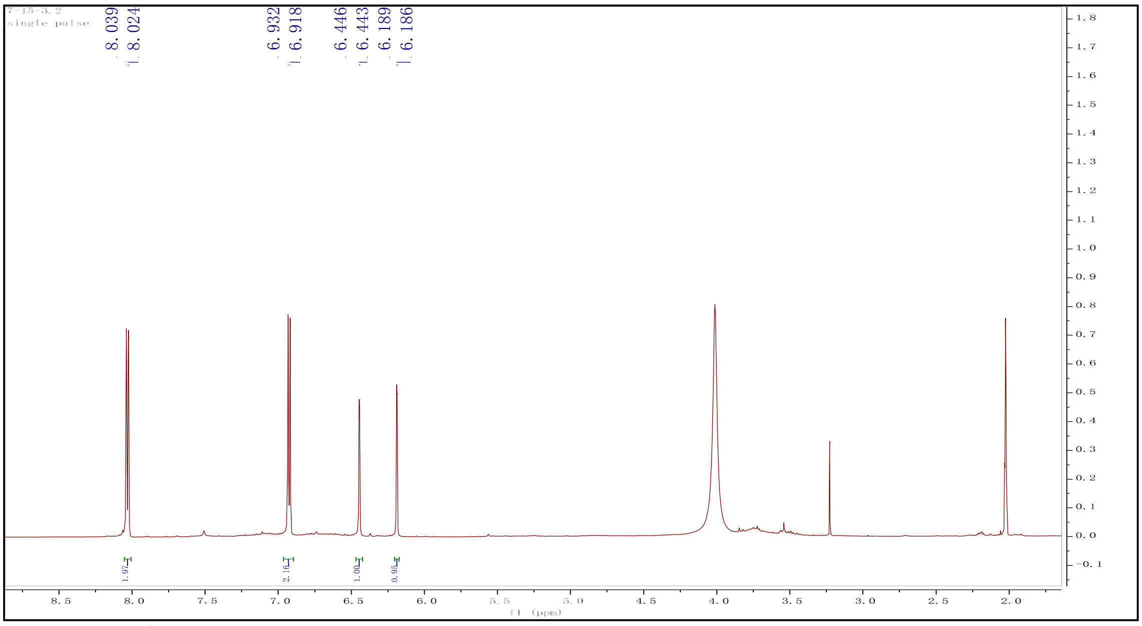

Figure S29.^{1}H-NMR spectrum (in 600 MHz) of compound **12** in $(CD_3OD)_2CO$- d_6 + D_2O

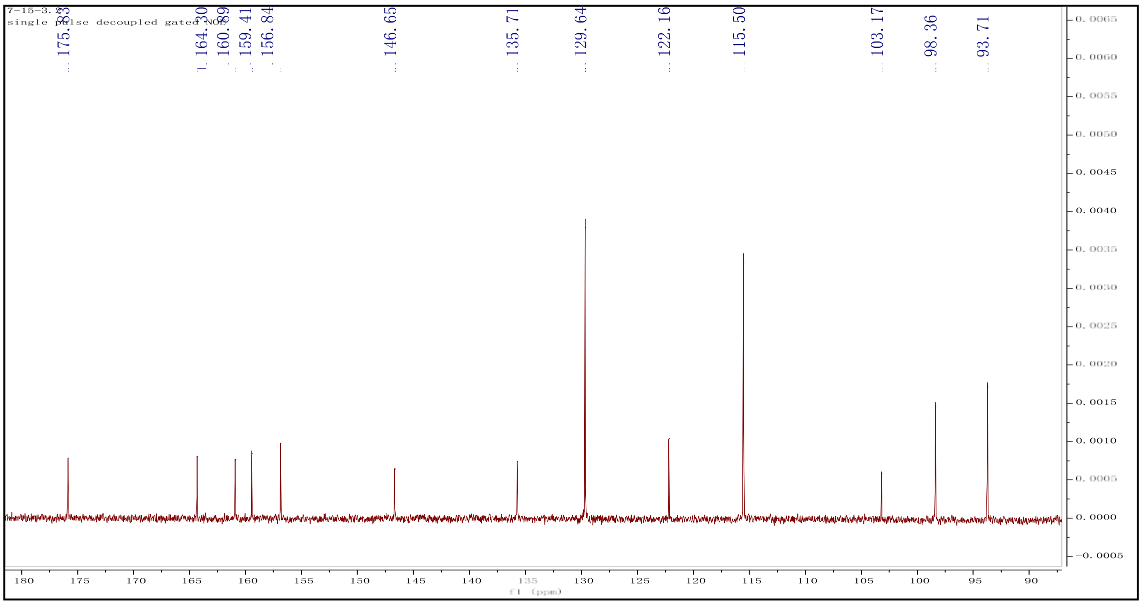

Figure S30.^{13}C-NMR spectrum (in 150 MHz) of compound **12** in $(CD_3OD)_2CO$- d_6 + D_2O

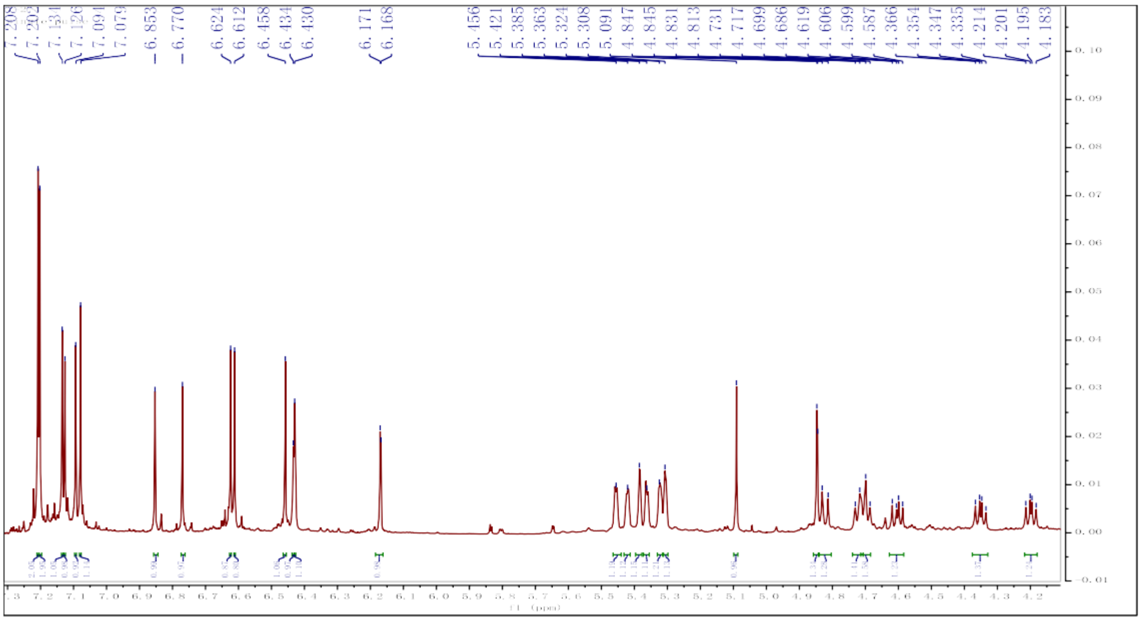

Figure S31.^{1}H-NMR spectrum (in 600 MHz) of compound **13** in $(CD_3OD)_2CO$- d_6 + D_2O

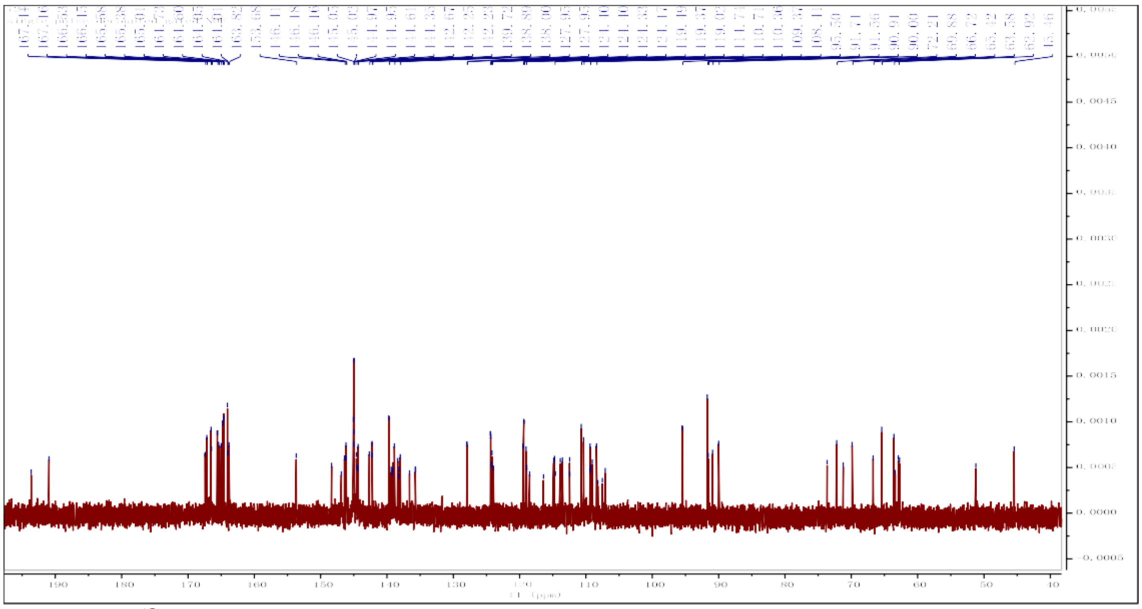

Figure S32. ^{13}C-NMR spectrum (in 150 MHz) of compound **13** in $(CD_3OD)_2CO$- d_6 + D_2O

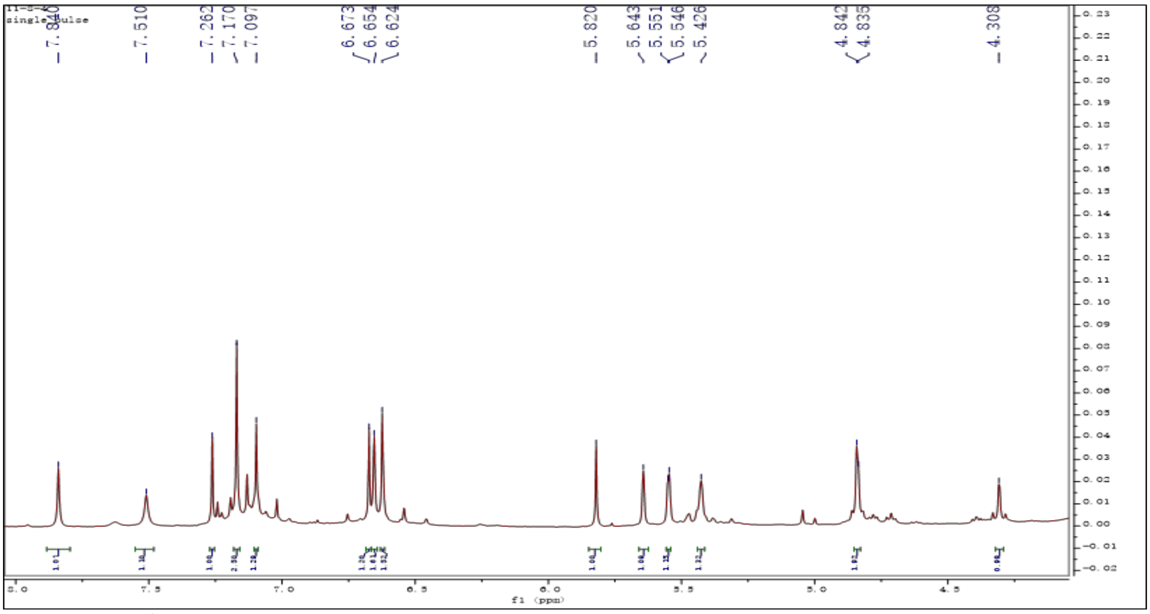

Figure S33. ^{1}H-NMR spectrum (in 600 MHz) of compound **14** in $(CD_3OD)_2CO$- d_6 + D_2O

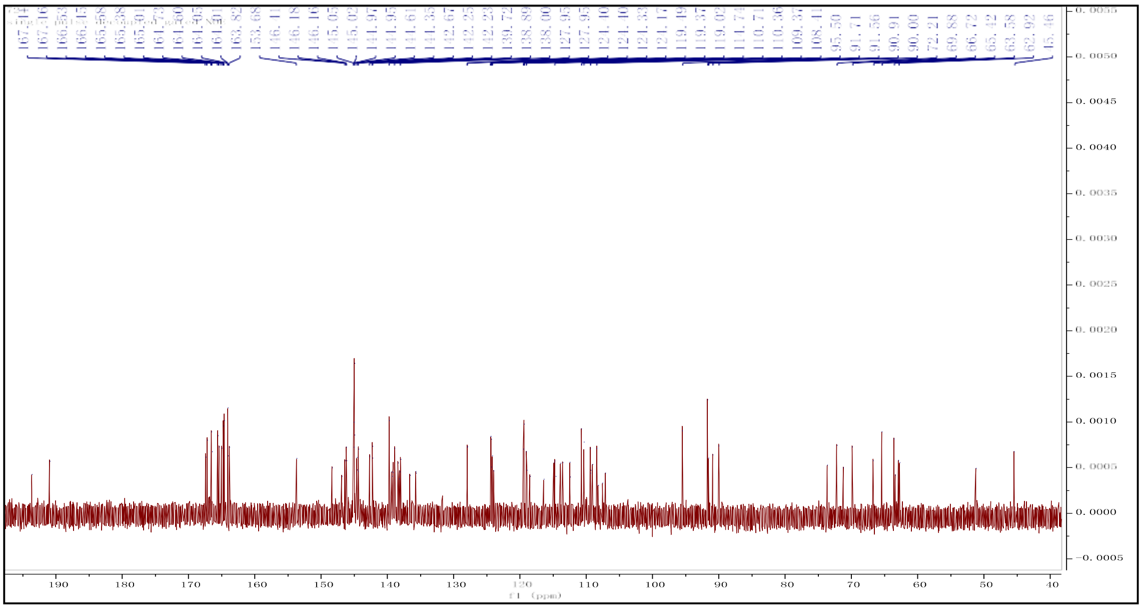

Figure S34.^{13}C-NMR spectrum (in 150 MHz) of compound **14** in $(CD_3OD)_2CO$- d_6 + D_2O

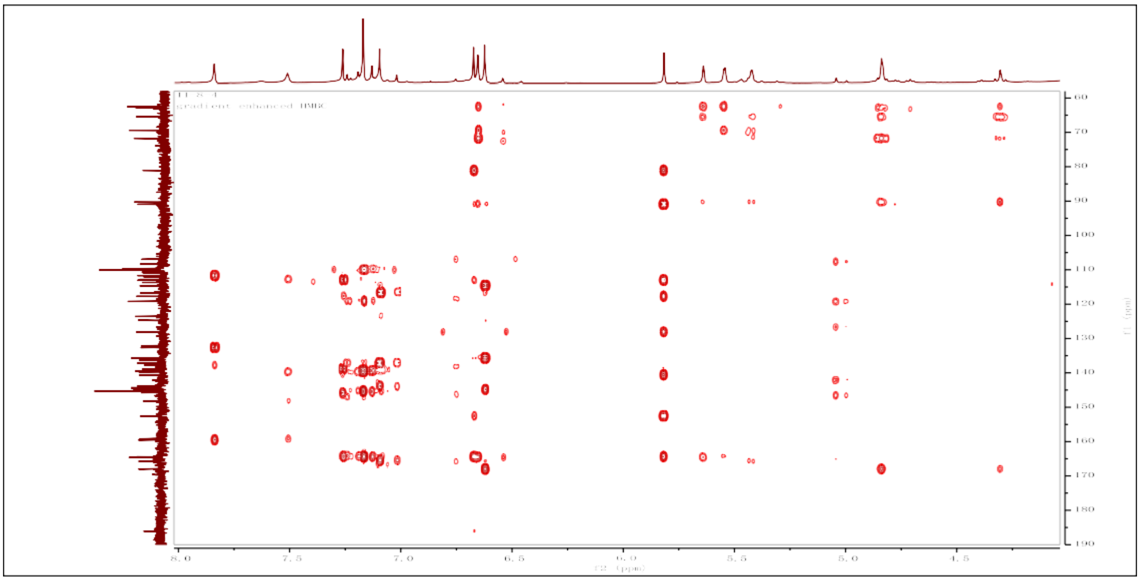

Figure S35. HMBC spectrum of compound **14** in $(CD_3OD)_2CO$- d_6 + D_2O

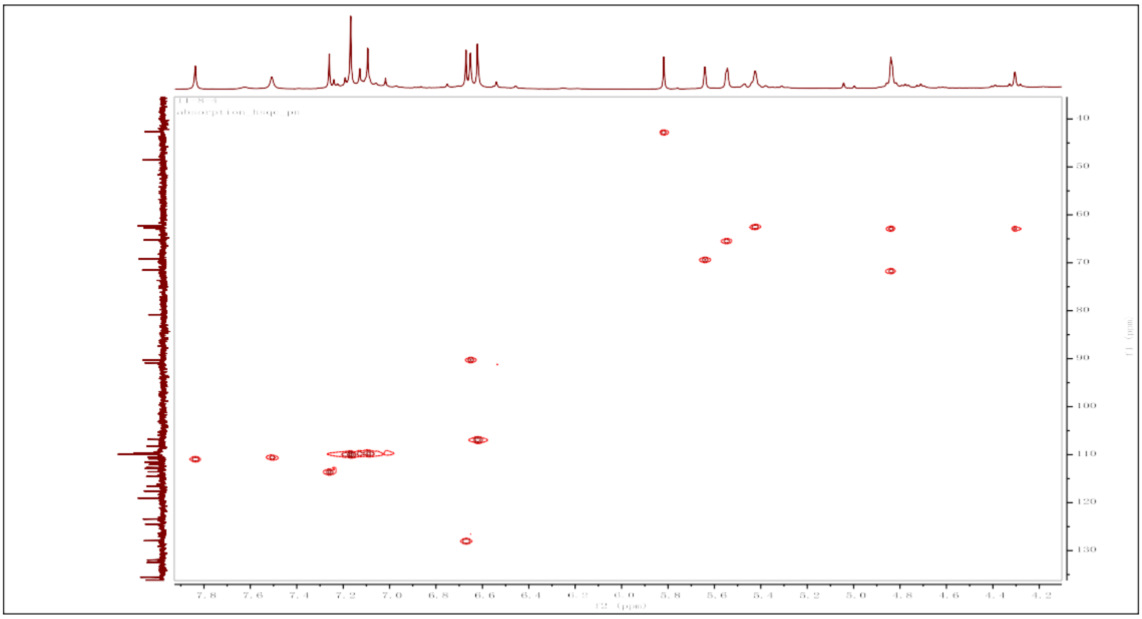

Figure S36. HSQC spectrum of compound **14** in $(CD_3OD)_2CO$- d_6 + D_2O

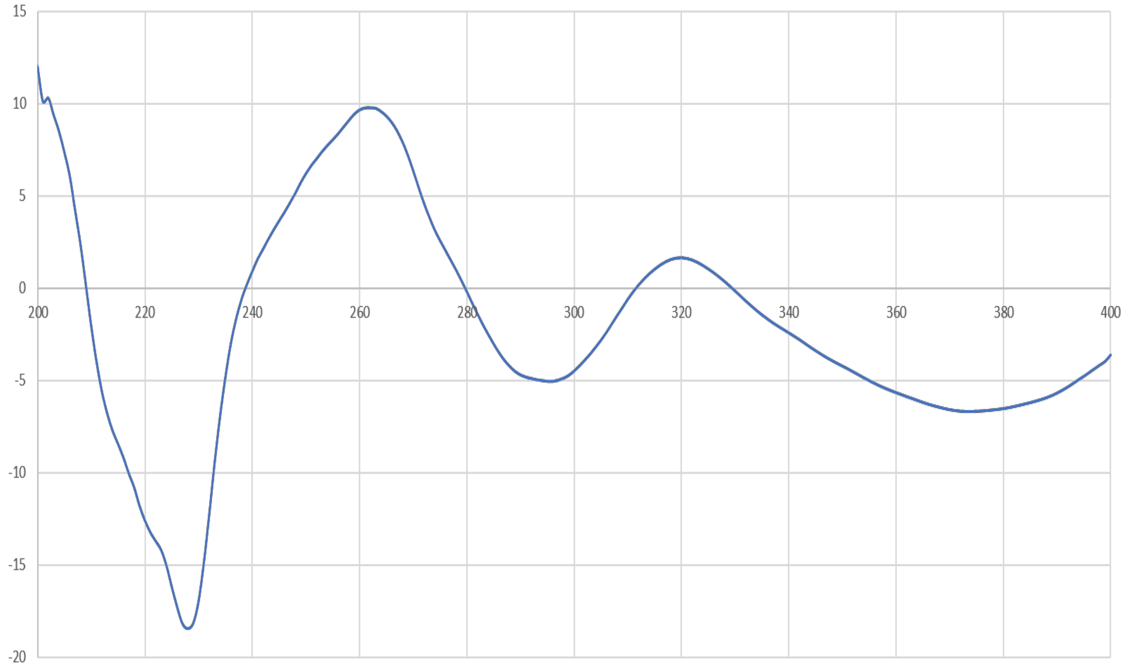

Figure S37. Circular dichroism (CD) spectrum of compound **14** (Methanol)

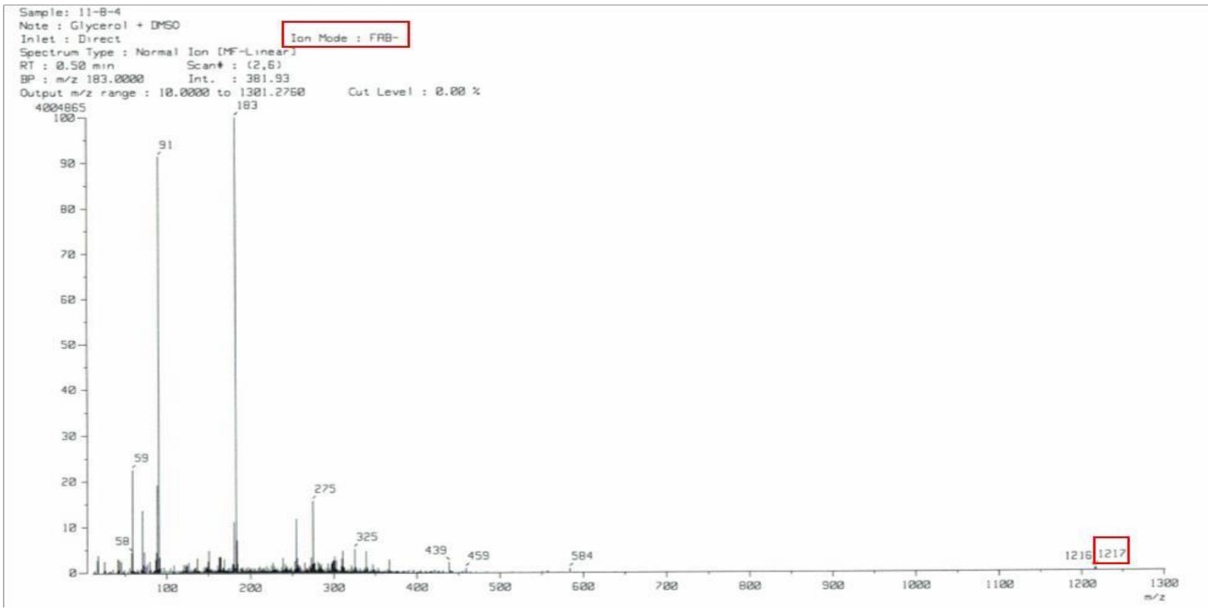

Figure S38. MS spectrum of **14**
